# Paracrine Secreted Frizzled-Related Protein 4 Inhibits Melanocytes Differentiation in Hair Follicle

**DOI:** 10.1155/2017/2857478

**Published:** 2017-02-27

**Authors:** Haiying Guo, Mingxing Lei, Yuhong Li, Yingxin Liu, Yinhong Tang, Yizhan Xing, Fang Deng, Ke Yang

**Affiliations:** ^1^Department of Cell Biology, Third Military Medical University, Chongqing 400038, China; ^2^“111” Project Laboratory of Biomechanics and Tissue Repair and Key Laboratory of Biorheological Science and Technology of Ministry of Education, College of Bioengineering, Chongqing University, Chongqing 400044, China; ^3^Chongqing Stem Cell Therapy Engineering Technical Center, Children's Hospital of Chongqing Medical University, Chongqing 400014, China

## Abstract

Wnt signaling plays crucial role in regulating melanocyte stem cells/melanocyte differentiation in the hair follicle. However, how the Wnt signaling is balanced to be overactivated to control follicular melanocytes behavior remains unknown. Here, by using immunofluorescence staining, we showed that secreted frizzled-related protein 4 (sFRP4) is preferentially expressed in the skin epidermal cells rather than in melanocytes. By overexpression of sFRP4 in skin cells in vivo and in vitro, we found that sFRP4 attenuates activation of Wnt signaling, resulting in decrease of melanocytes differentiation in the regenerating hair follicle. Our findings unveiled a new regulator that involves modulating melanocytes differentiation through a paracrine mechanism in hair follicle, supplying a hope for potential therapeutic application to treat skin pigmentation disorders.

## 1. Introduction

Melanocytes in the epidermis and hair follicle synthesize melanin granules to pigment skin and hairs. Melanocyte shows cyclic melanogenic activity coinciding with hair cycling, including telogen, anagen, and categen [1, 2]. When the hair cycle transitions from telogen to anagen, melanocyte stem cells (McSCs), which reside in the bulge area and secondary hair germ (sHG) of the hair follicle, differentiate into transient amplified melanocytes. These cells migrate into the newly formed hair bulb, becoming pigment-producing melanocytes [3–5]. During the anagen IIIa stage of the hair cycle, melanocytes synthesize melanin granules which are then transferred to the surrounding epidermal keratinocytes that give rise to the inner layers of the hair shaft, leading to pigmented hair formation [1, 2]. During catagen, melanin synthesis is ceased, and melanocytes are eliminated through cell apoptosis [6, 7], leaving the quiescent MSCs but not amplifying or differentiated melanocytes in bulge and sHG of hair follicles during telogen [7–9]. Besides, exogen and kenogen are independent cycle phases found in human and mouse hair follicle. The club hair fiber is shed during exogen, which is coupled with the next anagen phase [10, 11]. The follicle may keep empty under certain conditions such as androgenetic alopecia, leading to a phase called kenogen [12].

Wnt/*β*-catenin signaling pathway [13, 14] plays important roles in melanocyte development and differentiation. Mice deficient in Wnt1 and Wnt3a, or their downstream effector *β*-catenin, fail to generate melanocytes [15, 16]. On the contrary, overexpression of Wnt1, Wnt3a, or *β*-catenin to the cultured mouse neural crest cells results in expansion and differentiation of melanocyte [17, 18]. Our recent work also showed that Wnt3a and Wnt10b contribute to promoting the melanogenesis of melanocytes, whereas Wnt5a inhibits the melanogenesis [19–23]. However, how the Wnt signaling is balanced to control melanocytes differentiation in the hair follicle needs further investigation.

sFRP4 is a glycoprotein that acts as an antagonist of Wnt signaling. Previous studies show that sFRP4 is a modulator of cell differentiation and apoptosis in many tissues, such as mammary gland, ovary, and heart [24–26]. sFRP4 is also expressed in skin epidermis and adipose tissue in human and mouse [27, 28]. Several studies have demonstrated the importance of sFRP4 in homeostasis of skin tissues [27, 29–31]. As a Wnt inhibitor, sFRP4 functions as an extrafollicular modulator on coordinating the hair follicle cycling behavior [28, 30, 32]. Overexpression of sFRP4 reduces the proliferation of cultured human keratinocytes [27, 29] but promotes differentiation and apoptosis of human keratinocytes in vitro [27]. These studies suggest that sFRP4 plays a wide role in maintaining homeostasis of skin epidermal keratinocytes. As the crucial function of Wnt signaling on regulating melanocytes behavior, we infer that sFRP4 may also be involved in regulating melanocytes activity. To test this, we first detected sFRP4 expression in mouse hair follicles. Then, by overexpression of sFRP4 in skin cells in vivo and in vitro, we also investigated the role of sFRP4 in regulating melanocytes behaviors and found that sFRP4 inhibits the melanogenesis of melanocytes through antagonizing Wnt signaling.

## 2. Materials and Methods

### 2.1. Animals and Skin Samples

Dct-LacZ transgenic mice were kindly provided by Professor Ian Jackson [33]. Mouse skin samples with hair follicles representing catagen, telogen, early anagen, and mid-anagen were collected from 30-, 33-, 45-, and 56-day-old C57BL/6J mice. Mice were housed in the Animal Center of Third Military Medical University. The number of the permission is SYXK-PLA-20120031. All the animal-related procedures were in strict accordance with the approved institutional animal care and maintenance protocols.

Human skin samples were obtained from 30–40-year-old donors. Formalin-fixed, paraffin-embedded melanoma tissues were received from Xinqiao Hospital, the Third Military Medical University. The research was approved by the ethics board at Third Military Medical University.

### 2.2. Immunofluorescence

Immunostaining was performed on 5 *μ*m sections from tissue samples. Sections were dewaxed, rehydrated, and boiled in citrate buffer solution [34, 35]. After blocking, sections were incubated with the following primary antibodies: goat anti-sFRP4 (1 : 100, Abcam, Cambridge, USA), goat anti-TRP2 (1 : 100, Santa Cruz, City of Santa Cruz, CA, USA), mouse anti-AE13 (1 : 100, Santa Cruz, City of Santa Cruz, CA, USA), mouse anti-AE15 (1 : 100, Abcam, Cambridge, USA), rabbit anti- TRP1 (1 : 100, Santa Cruz, City of Santa Cruz, CA, USA), rabbit anti-*β*-catenin (1 : 200, Abcam, Cambridge, USA; 1 : 100, Beyotime, Beijing, China), and rabbit anti-PCNA (1 : 100, Abcam, Cambridge, USA). Alexa Fluor 488 (Invitrogen, Carlsbad, CA, USA) and CY3 (Beyotime, Nantong, China) were used as secondary antibodies. Finally, sections were counterstained with 4′,6-diamidino-2-phenylindole (DAPI) for nuclei visualization.

### 2.3. Intradermal Injection

To induce synchronized hair cycle, the hairs on the dorsal skin of 7-week-old mice were depilated as previously described [36, 37]. 25 *μ*L recombinant sFRP4 (50 *μ*g/mL, R&D Systems, Minneapolis, MN, USA) or PBS (control) was intradermally injected into the dorsal skin after depilation. Then the treated skin samples were harvested and prepared for analysis of histology or immunostaining.

### 2.4. X-Gal Staining

For X-gal staining, the samples were prefixed in 4% paraformaldehyde at 4°C for 1 hour, then stained with X-gal staining solution (Beyotime, Nantong, China) at room temperature for 24 h, and postfixed at 4°C overnight. After dehydration and embedding in paraffin, the skin samples were sliced into 5 *μ*m sections, which were then dewaxed, rehydrated, and stained with hematoxylin for nuclei visualization.

### 2.5. Cell Culture

The JB6Cl30-7b (JB6) cell line derived from primary cultures of neonatal mouse epidermal cells was purchased from ATCC (ATCC®CRL-2007™). The immortalized cell line of melanocyte progenitor, iMC23, was derived from neonatal mouse epidermis [38]. The cells were cultured in DMEM (high glucose; Hyclone, Waltham, MA, USA) supplemented with 10% fetal bovine serum (FBS; Gibco, Grand Island, NY, USA) and cultivated in an atmosphere containing 5% CO_2_ in air at 37°C.

### 2.6. Adenovirus Amplification and Infection

Adenoviruses including AdWnt3a, AdsFRP4, and AdGFP were gifted from Dr. Tong-Chuan He (University of Chicago). The adenoviruses were propagated in HEK293 cells as previously described [39]. After being purified by cesium chloride gradients, adenoviruses were dialyzed into storage buffer. The titers were adjusted with the storage buffer to be 10^8^ PFU/mL.

JB6 cells were plated onto the upper chamber of transwells, infected with AdsFRP4, AdWnt3a, and AdGFP (control) separately, or coinfected with AdsFRP4 and AdWnt3a. After 12 h incubation, the lower chambers were seeded with iMC23 cells. iMC23 cells and JB6 cells were cocultured for 72 h, then iMC23 cells were harvested to perform the tyrosinase activity assay or Masson-Fontana silver staining assay.

### 2.7. Tyrosinase Activity Assay

Cells were treated according to the aforementioned method. Tyrosinase activity assays were performed as previously described [21]. iMC23 cells were lysed with 1% Triton X-100/PBS at −80°C for 30 min and then thawed at 37°C. After centrifugation, 50 *μ*L supernatant was transferred into 96-well plates and added with 10 *μ*L L-DOPA (2 mg/mL; Sigma, St. Louis, MO, USA) per well. The absorbance was measured at 490 nm after the samples were incubated at 37°C for 2 hours. The relative activity of tyrosinase equals (OD group X − OD blank)/(OD control − OD blank) × 100%.

### 2.8. Masson-Fontana Silver Staining Assay

Cells were treated according to the aforementioned method. iMC23 cells were fixed with ice-cold acetone for 10 min and stained with a silver-ammonia solution (MYM Biological Technology Company, Beijing, China) at 37°C for 1 h. Finally, the cells were rinsed and the images were captured using a phase-contrast microscope.

### 2.9. Western Blot Analysis

Western blots were done based on previous descriptions [40–42]. Cell lysates were separated by 10% SDS-PAGE and transferred onto PVDF membranes. The membranes were incubated with antibodies: rabbit anti-TRP1 (1 : 1000, Santa Cruz, City of Santa Cruz, CA, USA), goat anti-TRP2 (1 : 1000, Santa Cruz, City of Santa Cruz, CA, USA), rabbit anti-tyrosinase (1 : 1000, Bioworld, St. Louis Park, MN, USA), and rabbit anti-*β*-catenin (1 : 1000, Abcam, Cambridge, USA) at 4°C overnight. Blots were then incubated with HRP-conjugated secondary antibody (Invitrogen, Carlsbad, CA, USA) for 1 hour. Bands were visualized on the membranes using an ECL western blotting detection system.

### 2.10. Statistical Analysis

Data were presented as a mean ± SD of three independent experiments. Statistical analyses were evaluated using *t*-test and *p* < 0.05 was considered statistically significant.

## 3. Results

### 3.1. sFRP4 Expression in Cyclic Hair Follicles

**Figure 1:**
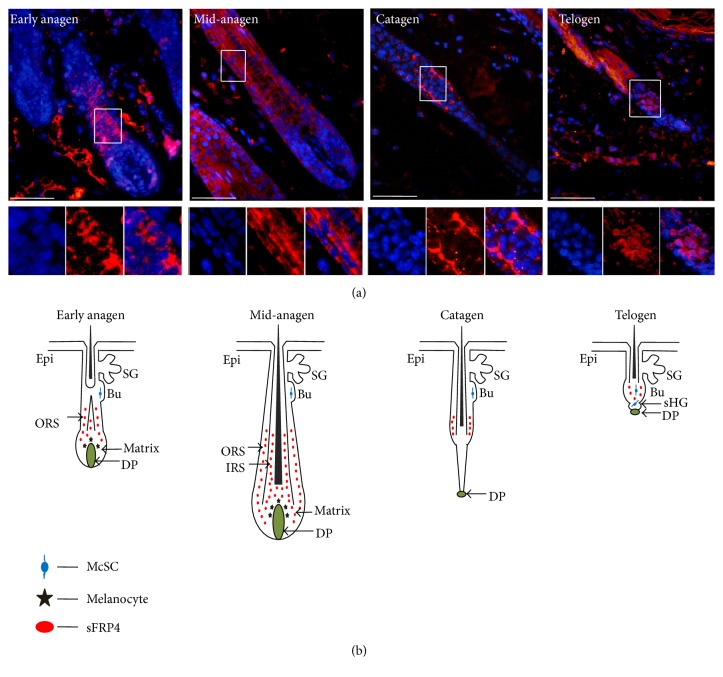
The expression pattern of sFRP4 in mouse hair follicle during hair cycling. (a) Immunostaining shows sFRP4 expression in dorsal skin of C57BL/6 mice at P30 (early anagen), P35 (mid-anagen), P45 (catagen), and P56 (telogen). The lower panels show the higher power view of the upper panels. (b) Schematic of sFRP4 expression pattern in hair follicle. Epi: epidermis; SG: sebaceous gland; Bu: bulge; DP: dermal papilla; HG: hair germ; IRS: inner root sheath; ORS: outer root sheath. Bars: 50 *μ*m.

We first detected the expression pattern of sFRP4 in mouse dorsal skin by immunofluorescence (Figures 1(a) and 1(b) and Figures s1-s2, in Supplementary Material available online at https://doi.org/10.1155/2017/2857478). sFRP4 is strongly expressed in the precursors of the inner root sheath (IRS) in hair follicles and the interfollicular dermal region during early anagen. In mid-anagen, sFRP4 is expressed in the IRS precursors, IRS, and outer root sheath (ORS) and weakly in the hair matrix. During catagen, sFRP4 is mainly expressed in the ORS above the epithelial strand and weakly in dermis. When hair follicles enter telogen, sFRP4 is expressed in the bulge of hair follicle and strongly expressed again in the dermal region surrounding the hair follicle.

### 3.2. sFRP4 Is Preferentially Expressed in Epithelial Cells but Not in Adjacent Melanocytes

**Figure 2:**
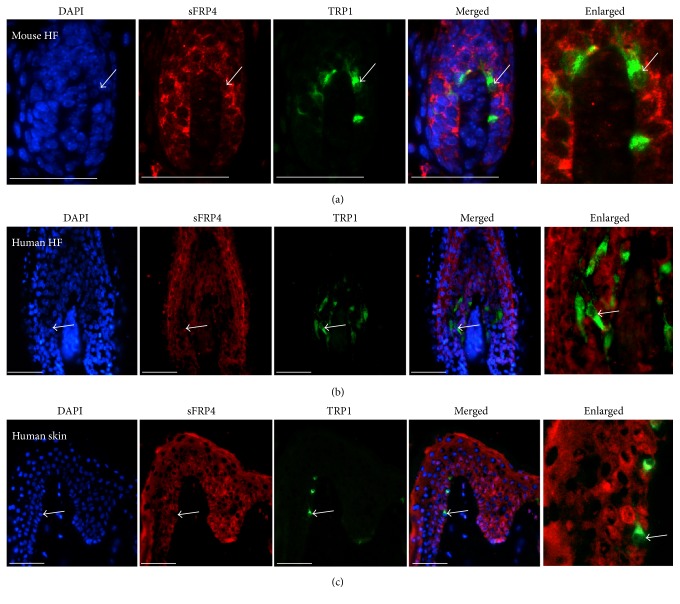
The coexpression of sFRP4 and TRP1 in mouse and human HF and human skin. The right panel shows the enlarged view of the labeled area in the left panel. (a-b) sFRP4 is expressed in the hair matrix and HS precursors surrounding the TRP1 positive cells in both mouse (a) and human (b) HF. (c) sFRP4 is expressed in keratinocytes surrounding TRP1 positive cells in human skin. Bars: 50 um.

Our data showed that, in anagen hair follicles, sFRP4 is expressed in the IRS precursors and hair matrix region where also follicular melanocytes reside. We then performed double immunofluorescent staining for sFRP4 and TRP1 (a marker of melanocytes) [43] to determine whether sFRP4 is expressed in follicular melanocytes. Interestingly, we found that sFRP4 is restrictedly expressed in the hair matrix and IRS precursor but not expressed in melanocytes in mouse and human hair follicles (Figures 2(a) and 2(b)). We also examined the coexpression of sFRP4 and TRP1 in normal human skin epidermis and found that sFRP4 is also not expressed in melanocytes in human skin but in their neighboring epidermal cells (Figure 2(c)). In summary, these data suggested that sFRP4 is preferentially expressed in the skin epidermal cells rather than in melanocytes.

### 3.3. Overexpression of sFRP4 in Skin Reduces the Number of Follicular Melanocytes In Vivo

**Figure 3:**
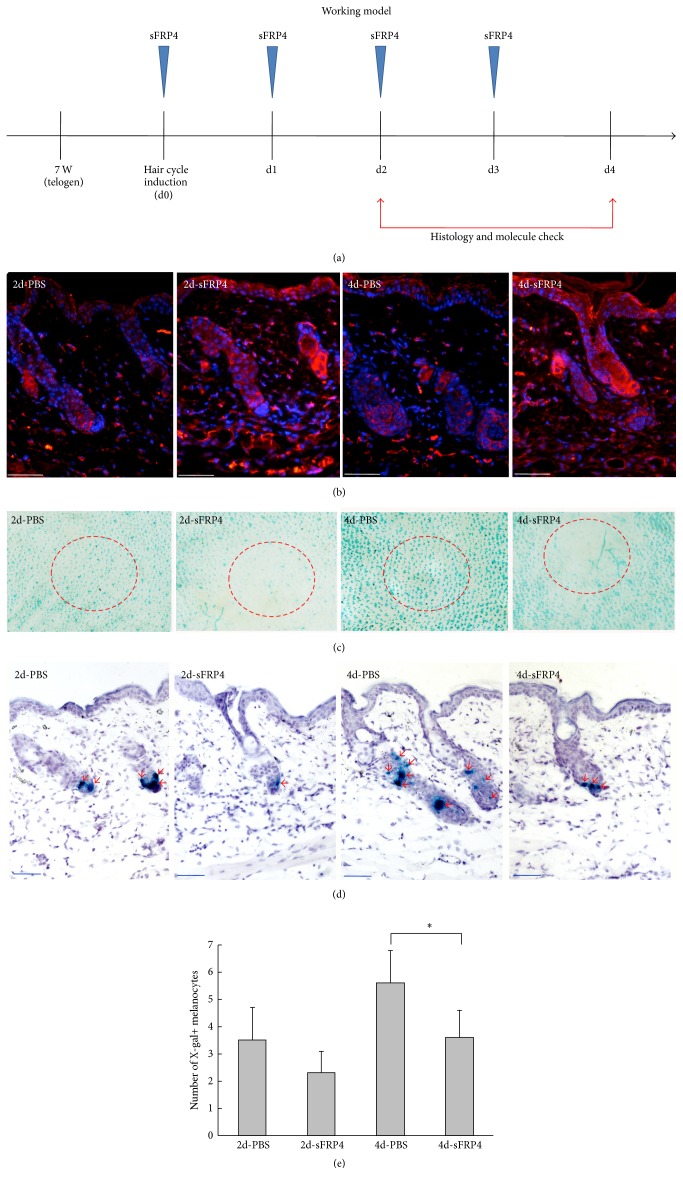
Effects of sFRP4 on melanocyte differentiation in vivo. (a) Schematic drawing showing the timing of multiple injections of sFRP4 protein, hair cycle events, and check points. Hair cycle initiation was induced by depilation of the back skin of 7-week-old Dct-LacZ mice. sFRP4 recombinant protein or PBS was administrated intracutaneously at days 0, 1, 2, and 3 after depilation. The histology and molecule check were performed 2 days and 4 days after intradermal injection of sFRP4 recombinant protein. (b) The sFRP4 expression was detected by immunofluorescence after PBS or sFRP4 treatment. (c) Whole-mount X-gal staining reveals the Dct-LacZ labeled McSCs and differentiated melanocytes. The dotted circle represents the injected region. (d) X-gal staining counterstained with H&E staining shows the LacZ-positive melanocytes in the hair follicles (red arrow, X-gal staining). (e) Statistical analysis reveals the number of LacZ-positive melanocytes is significantly decreased after sFRP4 treatment, when compared to the controls. Data are reported as average ± SD. ^*∗*^*p* < 0.05. Bars: 50 *μ*m.

We then investigated the role of sFRP4 in follicular melanocytes in vivo. We depilated 7-week-old Dct-LacZ transgenic mice (in the second telogen phase) to induce synchronized anagen reentry of hair follicles, which also results in activation of McSCs [44, 45]. Immediately after depilation, we continuously administrated sFRP4 recombination protein or PBS (control) into the dorsal skin once a day for 4 days (Figure 3(a)). The skin samples were harvested different days after injection (Figure s3). Immunostaining confirmed that the expression of sFRP4 protein was increased in dermis, epidermis, and hair follicles (Figure 3(b)). X-gal staining of skin sections and whole-mount tissues shows that the number of melanocytes was significantly decreased 4 days after injection of sFRP4 recombinant protein, compared to the control group (Figures 3(c)–3(e)). However, the skin eventually turned black when sFRP4 administration is ceased (Figure s3), indicating the temporal effect of overexpressed-sFRP4 on melanocyte differentiation.

**Figure 4:**
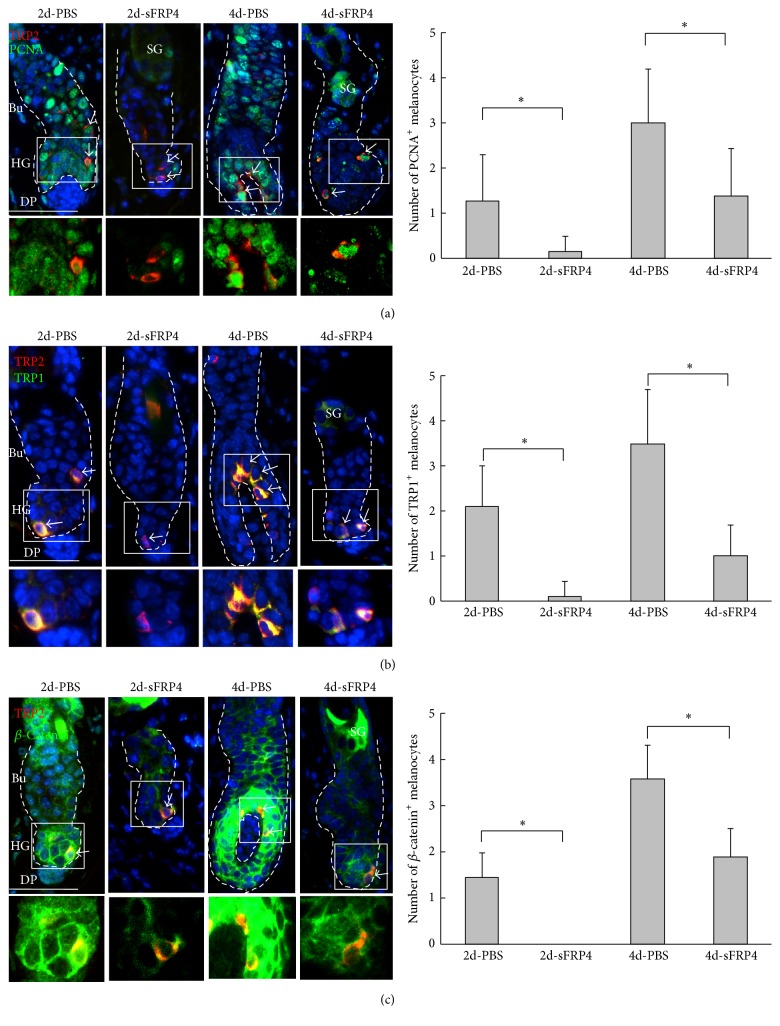
Immunofluorescence staining shows proliferation and differentiation of melanocytes after sFRP4 treatment. (a) Double staining shows colocalization of PCNA and TRP2 (white arrow, PCNA), and statistical chart reveals PCNA-positive proliferative cells in the hair follicles after sFRP4 or PBS treatment. (b) Double staining shows colocalization of TRP1 and TRP2 (white arrow, TRP1), and statistical chart reveals TRP1 expression in the hair follicles after sFRP4 or PBS treatment. (c) Double staining shows colocalization of *β*-catenin and TRP2 (white arrow, *β*-catenin), and statistical chart reveals nuclear *β*-catenin expression in the hair follicles after sFRP4 or PBS treatment. Data are reported as average ± SD. ^*∗*^*p* < 0.05. Bars: 50 um.

We further examined the cell proliferation (PCNA) and differentiation (AE13 and AE15) after sFRP4 treatment. The result showed that the proliferated and differentiated cells are largely reduced in hair follicles after the sFRP4 treatment for 2 and 4 days (Figures s4-s5), suggesting that sFPR4 also influences hair cycling, which was demonstrated by the previous study [30]. Interestingly, by double staining of TRP2 and PCNA, we observed that the proliferating melanocytes are also reduced after sFRP4 treatment (Figure 4(a)). These data indicate that sFRP4 inhibits melanocyte proliferation in hair follicles at early days after depilation.

### 3.4. sFRP4 Inhibits Wnt-Induced Differentiation of Follicular Melanocytes In Vivo

To investigate the effect of sFRP4 on the differentiation of McSCs/melanocytes, we examined the expression of TRP1, which is a differentiation marker of McSCs/melanocytes [43]. Two days after depilation, TRP1 was detected in the TRP2-positive melanocytes at the sHG region in the PBS-treated group, but not in the sFRP4-treated group. Then, 4 days after depilation, both TRP2 and TRP1 were expressed in the hair follicles that were treated with sFRP4 or PBS (Figure 4(b)). However, the numbers of TRP1-positive melanocytes were significantly decreased after sFRP4 treatment, when compared to the PBS treatment group (*p* < 0.05). These data further suggest that sFRP4 inhibits McSCs differentiation and maintains melanocytes in an undifferentiated state.

To further explore the mechanisms of how sFRP4 inhibits McSCs differentiation in hair follicle, we detected the expression of *β*-catenin which is a key effector of Wnt signaling. We observed that nuclear *β*-catenin was present in TRP2-positive melanocytes in the PBS-treated hair follicles, but not in the sFRP4-treated hair follicles 2 days after depilation. Then, 4 days after depilation, *β*-catenin positive cells were increased in hair matrix and TRP2-positive melanocytes in the PBS-treated hair follicles. However, the number of *β*-catenin-positive melanocytes was significantly reduced in the hair follicles showing a delayed anagen reentry 4 days after sFRP4 treatment (*p* < 0.05, Figure 4(c)). These results suggest that sFRP4 inhibits melanocyte differentiation by inhibition of the canonical Wnt/*β*-catenin signaling pathway.

### 3.5. sFRP4 Reverse Wnt3a-Induced Melanogenesis In Vitro

**Figure 5:**
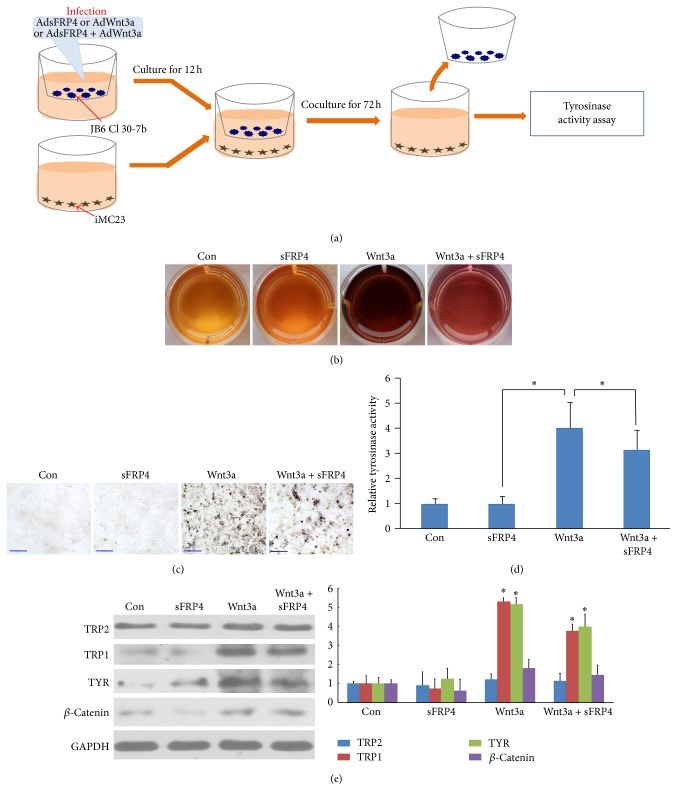
Effects of sFRP4 on Wnt3a-induced differentiation in melanocytes in vitro. (a) JB6 epithelial cells were infected with AdsFRP4, AdWnt3a, and AdGFP, respectively, or coinfected with AdWnt3a and AdsFRP4. Then JB6 cells and iMC23 melanocyte progenitors were cocultured. (b) Direct observation of melanogenesis of iMC23 cells 5 days after being cocultured with JB6 cells. (c) Masson-Fontana staining reveals the melanin granules formation. Bars: 100 um. (d) Tyrosinase activity assay of iMC23 cells 3 days after coculture. The values indicate the mean of three independent experiments ± SD. ^*∗*^*p* < 0.05. (e) Western blot and statistical analysis reveal the protein expression levels of TRP2, TRP1, tyrosinase, and *β*-catenin in different treatment groups.

Wnt ligands expressed by epithelial cells can activate Wnt signaling in neighboring melanocytes in early anagen hair follicle [3]. To further confirm whether sFRP4 secreted by epithelial cells can influence neighboring melanocytes differentiation through inhibiting Wnt signaling, we cocultured AdWnt3a- and/or AdsFRP4-infected JB6 epithelial cells with iMC23 melanocyte progenitor cells (Figure 5(a)). Three days after coculture, tyrosinase activity assay which reflects melanogenesis was performed (Figure 5(d)). The results showed that the tyrosinase activity was significantly enhanced in Wnt3a-treated group, when compared to the control group which was treated with AdGFP. Nevertheless, tyrosinase activity was significantly decreased when AdsFRP4 was added to the AdWnt3a-treated group (*p* < 0.05). The melanin production was also directly observed through the pigmented cell pellets due to melanogenesis. The formation of melanin was reduced in Wnt3a + sFRP4-treated group compared to Wnt3a-treated group (Figure 5(b)). This was further confirmed by Masson-Fontana silver staining (Figure 5(c)). We further tested the protein expression of TRP1, TRP2, tyrosinase, and *β*-catenin in different experimental groups by western blots. The results showed that the expression of these differentiation-related genes was decreased in Wnt3a + sFRP4-treated group compared to the Wnt3a-treated group (Figure 5(e)).

Taken together, these results suggest that sFRP4 inhibits the differentiation of melanocytes through abrogating Wnt3a-induced Wnt/*β*-catenin signaling in vitro.

## 4. Discussion

**Figure 6:**
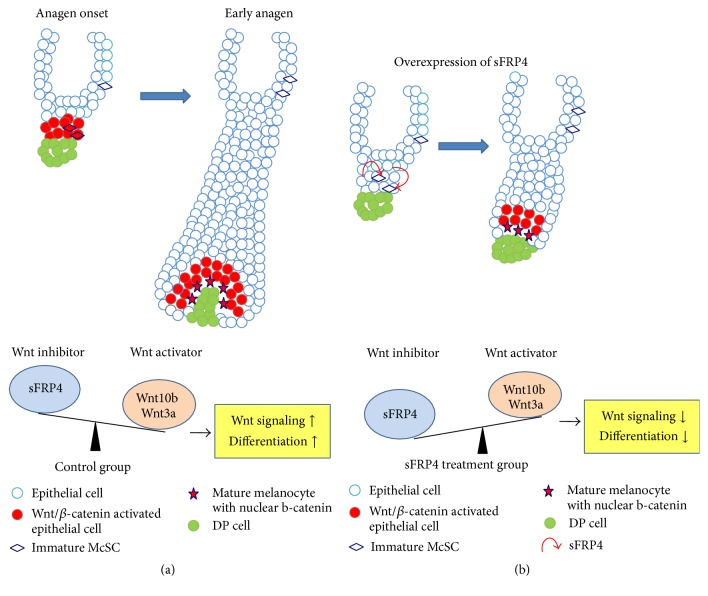
Schematic of the mechanism of how sFRP4 functions on melanocyte differentiation. In the control group (a), there are more Wnt activators (such as Wnt10b, 3a) and less Wnt inhibitors (such as sFRP4) during anagen onset to early anagen phase, leading to melanocytes differentiation. However, the balance is tilted when sFPR4 is overexpressed (b). More Wnt inhibitors cause decreased melanocytes differentiation.

One of the most important theories in hair follicle biology is the indirect regulation of cell behaviors by the secreted factors from the microenvironment niche and macroenvironment tissues [46–49]. Activation of McSCs during hair regeneration was a paradigm [45]. As crucial regulatory factors, Wnt signaling plays vital roles in the differentiation of McSCs/melanocytes in hair follicles [3, 20, 21, 23]. Activated Wnt signaling drives McSCs/melanocytes undergoing differentiation in cyclic hair follicles [3, 20, 21, 23]. However, whether the environmental factors can antagonize the increasing expression of Wnt signaling-induced differentiation of melanocytes at anagen phase needs further investigation. Here we show that sFRP4 secreted by the neighboring epithelial cells inhibits melanocyte differentiation during hair cycling, via antagonizing activation of Wnt/*β*-catenin signaling pathway (Figure 6).

Previous studies showed that sFRP4 mRNA is mainly expressed in the extrafollicular dermal region [32]. By detecting the expression pattern of sFRP4 protein in the skin, we found that sFRP4 was not only expressed in the intrafollicular dermal region, but also expressed in the hair follicle during hair cycling. Interestingly, we found that sFRP4 is not expressed in the melanocytes, but in the surrounding keratinocytes. Through our functional data, we showed that keratinocytes-secreted sFRP4 inhibits differentiation of iMC23 melanocyte progenitors. This raises a key question: why does the hair follicle evolve such a paracrine rather than an autocrine mechanism to regulate melanocytes differentiation? First, as aforementioned, cells in the hair follicle are more or less influenced by their neighboring cells during the hair cycling. In addition to inhibiting melanocytes differentiation reported in the present study, sFPR4 also has other functions such as delaying hair cycling [30]. So it is reasonable to speculate that melanocytes differentiation influenced by paracrine sFPR4 is a passive biological process. Through antagonizing Wnt/*β*-catenin, sFPR4 further regulates melanocytes differentiation. Second, unlike the inner bulge cells, which are not responsive for the local or environmental stimuli [50], it looks like melanocytes are a group of cells that are susceptible to the environmental factors secreted by neighboring keratinocytes and fibroblasts [51, 52]. Third and most important, it is also reasonable to speculate that the skin evolves multiple ways to protect organism from environmental insult [47]. For example, the UV radiation induces melanocytes differentiation to prevent the skin from injury [53]. Thus, under normal conditions, the neighboring epithelia-secreted factors further serve to enhance the quiescent state of melanocytes to prevent them from overdifferentiation.

The melanocytes that are differentiated from McSCs migrate out from bulge region in anagen phase and maintain their melanogenesis ability, which is reported to be largely regulated by other factors, such as Wnts [3]. However, overactivation of Wnt signaling pathway induces aberrant differentiation of McSCs, leading to skin hyperpigmentation such as solar lentigo and premature hair graying [3, 53]. Also, continuous activation of *β*-catenin in melanocytes even causes melanoma formation in mice [54]. Thus, proper regulation of melanocyte behaviors requires proper Wnt signaling activity during hair cycling. Our study unveiled that sFRP4 is important for controlling the differentiation of melanocytes during hair cycling, through a paracrine mechanism.

sFRP4 is also largely involved in other pathways [55]. In the present study, we showed that overexpression of sFRP4 results in a decrease of Wnt/*β*-catenin signaling pathway activation, leading to less differentiation of melanocytes. This suggests that sFRP4 inhibits melanocytes differentiation through inhibiting Wnt/*β*-catenin signaling pathway. What is the exact mechanism in which sFRP4 inhibits Wnt/*β*-catenin signaling pathway to influence melanocytes differentiation? Our recent work showed that Wnt3a contributes to promoting melanocytes differentiation through increasing Wnt/*β*-catenin signaling pathway [21, 22]. It is also reported that sFRP4 abrogates Wnt3a-induced *β*-catenin to modulate the mammary differentiation in vitro [25]. By application of both sFRP4 and Wnt3a to the coculture system containing iMC23 melanocyte progenitors in the present study, we also verified that overexpressed sFRP4 in keratinocytes can antagonize Wnt3a-induced Wnt/*β*-catenin signaling pathway, resulting in a decrease of Wnt3a-induced differentiation.

In conclusion, our findings demonstrate a paracrine mechanism that is involved in melanocytes differentiation during hair cycling. As a Wnt antagonist, sFRP4 is expressed by keratinocytes and inhibits the differentiation of neighboring melanocytes via inhibiting Wnt/*β*-catenin signaling pathway. Importantly, our results provide future directions to investigate signaling mechanisms involving regulation between melanocytes and keratinocytes and also supply a hope for a potential clinical usage to treat skin pigmentation disorders.

## Supplementary Material

In Supplementary Figure 1 and 2, the expression pattern of sFRP4 in mouse dorsal skin was detected by immunofluorescence. In Supplementary Figure 3, the effect of sFRP4 on the pigmentation of hair follicle was detected. The result showed that the skin eventually turned black when sFRP4 administration was ceased. In Supplementary Figure 4, the effect of sFRP4 on the differentiation of hair follicle cells was examined. The result showed that the differentiated cells were reduced after sFRP4 treatment. In Supplementary Figure 5, the effect of sFRP4 on the proliferation of hair follicle cells was examined. The result showed that the proliferated cells were reduced after sFRP4 treatment.

## Abbreviations

Ad: Adenovirus
DP: Dermal papillae
HF: Hair follicle
IRS: Inner root sheath
ORS: Outer root sheath
sFRP4: Secreted frizzled-related protein 4
sHG: Second hair germ
McSC: Melanocyte stem cell
TRP1: Tyrosinase-related protein 1
TRP2: Tyrosinase-related protein 2
TYR: Tyrosinase
Wnt: Wingless-type MMTV integration site family
X-gal: 5-Bromo-4-chloro-3-indolyl-*β*-D-galactoside.
