# Supplementary material for: Paracrine Secreted Frizzled-Related Protein 4 Inhibits Melanocytes Differentiation in Hair Follicle
Source: Stem Cells Int. 2017 Feb 27;2017:2857478. doi: 10.1155/2017/2857478 (PMC5350338; doi:10.1155/2017/2857478)
Supplement: Supplementary file 1 — In Supplementary Figure 1 and 2, the expression pattern of sFRP4 in mouse dorsal skin was detected by immunofluorescence. In Supplementary Figure 3, the effect of sFRP4 on the pigmentation of hair follicle was detected. The result showed that the skin eventually turned black when sFRP4 administration was ceased. In Supplementary Figure 4, the effect of sFRP4 on the differentiation of hair follicle cells was examined. The result showed that the differentiated cells were reduced after sFRP4 treatment. In Supplementary Figure 5, the effect of sFRP4 on the proliferation of hair follicle cells was examined. The result showed that the proliferated cells were reduced after sFRP4 treatment. [file 2857478.f1.zip › Supplementary Materials and methods.docx]

**Supplementary Materials and methods**

**1. skin samples**

Mouse skin samples with hair follicles representing catagen, telogen, early-anagen and mid-anagen were collected from 30-, 33-, 45-, and 56- day-old C57BL/6J mice.

**2. Immunofluorescence**

Immunostaining was performed on 5 μm sections from tissue samples. Sections were dewaxed, rehydrated, and boiled in citrate buffer solution. After blocking, sections were incubated with the following primary antibodies: goat anti- sFRP4 (1 : 100, Abcam, Cambridge, USA), mouse anti-AE13 (1 :  100, Santa Cruz, City of Santa Cruz, CA, USA), mouse anti-AE 15 (1 : 100, Abcam, Cambridge, USA), rabbit anti- PCNA (1 : 100, Abcam, Cambridge, USA). Alexa Fluor 488 (Invitrogen, Carlsbad, CA,USA) and CY3 (Beyotime, Nantong, China) were used as secondary antibodies. Finally, sections were counterstained with 4^′^,6-diamidino-2-phenylindole (DAPI) for nuclei visualization.

**3. Intradermal injection**

To induce synchronized hair cycle, the hairs on the dorsal skin of 7-week-old mice were depilated. 25 μL recombinant sFRP4 (50 μg/mL, R&D Systems, Minneapolis, MN, USA) or PBS (control) was intradermally injected into the dorsal skin after depilation. Then the treated skin samples were observed different days after injection.

**Supplementary Figures and Legends**

Figure s1. The expression pattern of sFRP4 in mouse skin and cycling hair follicle. Immunostaining of sFRP4 in dorsal skin of C57BL/6 mice at P30 (early anagen), P35 (mid-anagen), P45 (catagen) and P56 (telogen). Bars = 50 μm.

Figure s2. Immunostaining of the dorsal skin of C57BL/6 mice at P35.Images show staining with an CY3 secondary Ab alone without sFRP4 primary antibody. The data shows no unspecific staining. Bars = 50 μm.

Figure s3. Effects of sFRP4 on pigmentation of mouse HF. Hair cycling was induced by depilation of the back skin of 7-week-old Dct-LacZ mice. sFRP4 recombinant protein or PBS was administrated intracutaneously after depilation. The mice were photographed7, 10, 13, 16 days and 30 days after intradermal injection of sFRP4.

Figure s4. Immunofluorescence staining shows differentiation of hair follicle cells after sFRP4 treatment. (a) The expression of AE13, characterizing keratins in the upper cortex and hair cuticle cells, was decreased in the hair follicles after sFRP4 treatment. (b) The expression of AE15, characterizing keratins in the IRS and medulla of hair shaft, was decreased in the hair follicles after sFRP4 treatment. Bars = 50 um

Figure s5. Immunofluorescence staining shows proliferation of hair follicle cells after sFRP4 treatment. The PCNA-positive proliferative cells in the hair follicles was decreased after sFRP4 treatment. Bars = 50 um
