# Supplementary figures and images for: Paracrine Secreted Frizzled-Related Protein 4 Inhibits Melanocytes Differentiation in Hair Follicle
Source: Stem Cells Int. 2017 Feb 27;2017:2857478. doi: 10.1155/2017/2857478 (PMC5350338; doi:10.1155/2017/2857478)

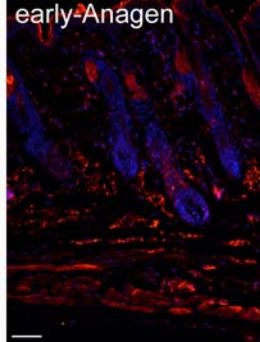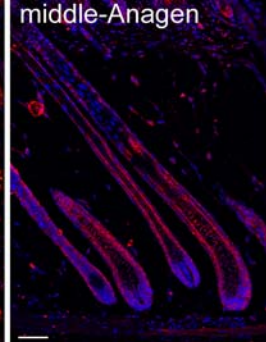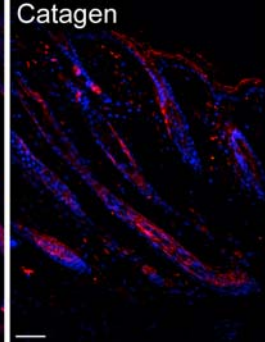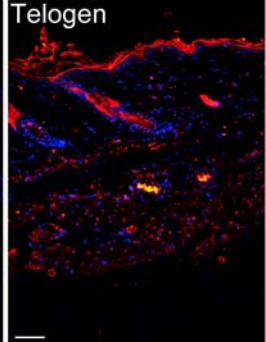

DAPI

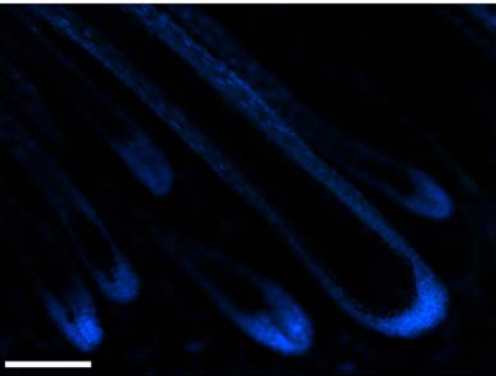

CY3

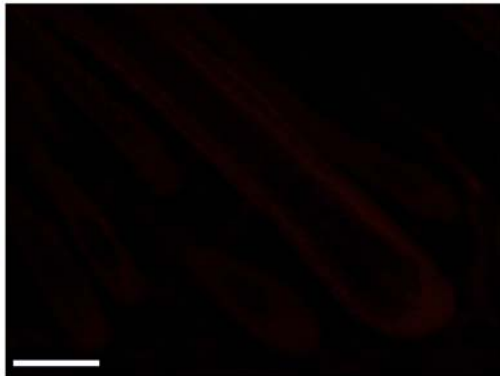

Merged

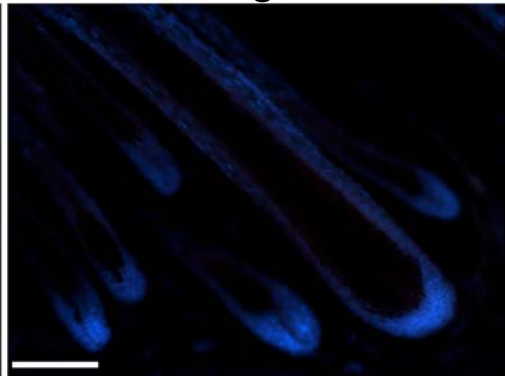

d7

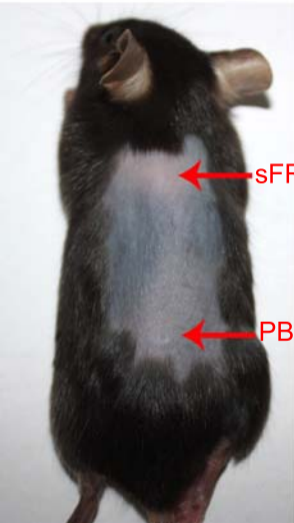

d10

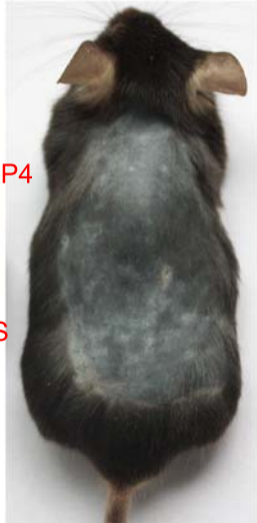

d13

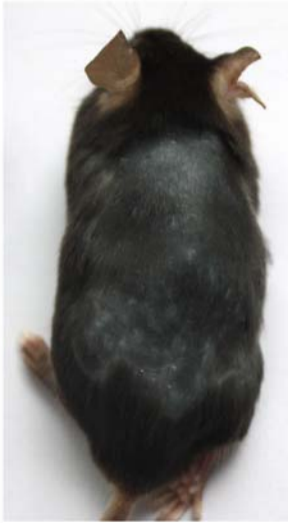

d16

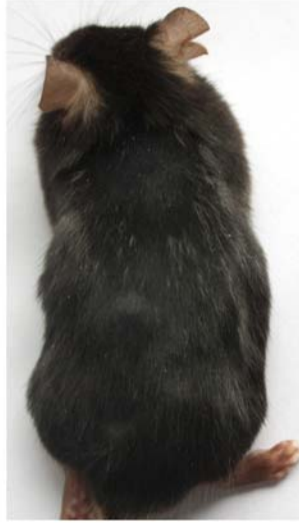

d30

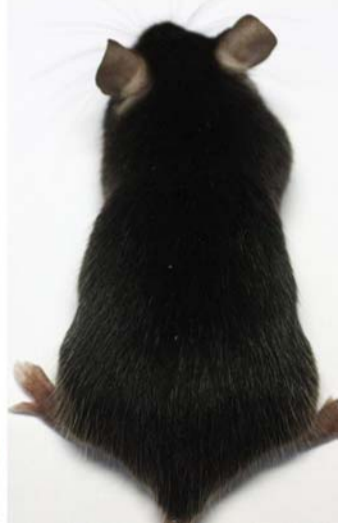

4d-PBS

4d-sFRP4

(a) AE13

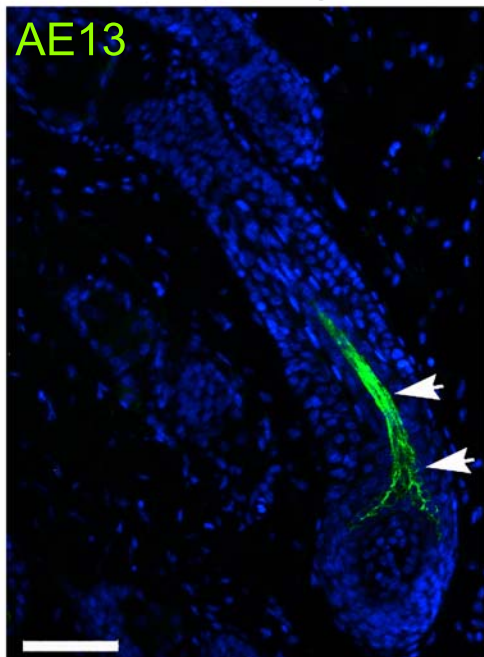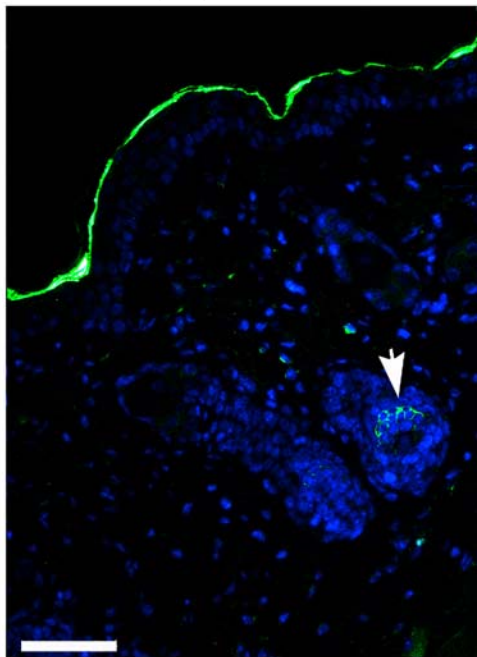

(b) AE15

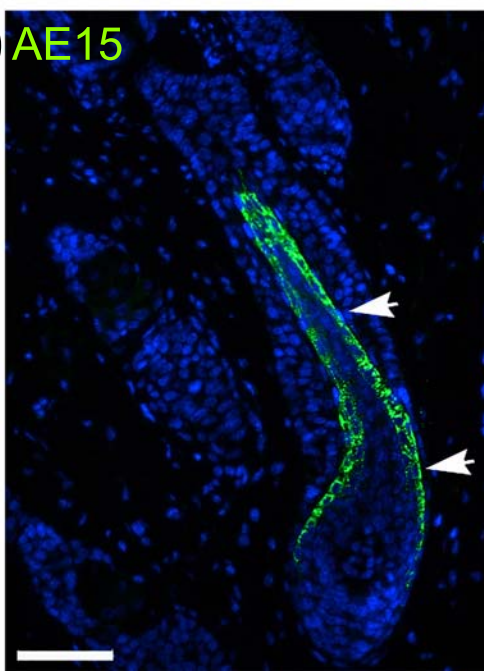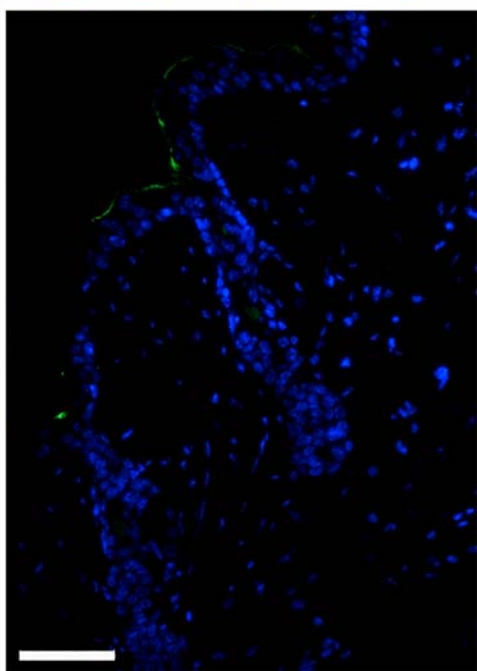

PBS

sFRP4

2d

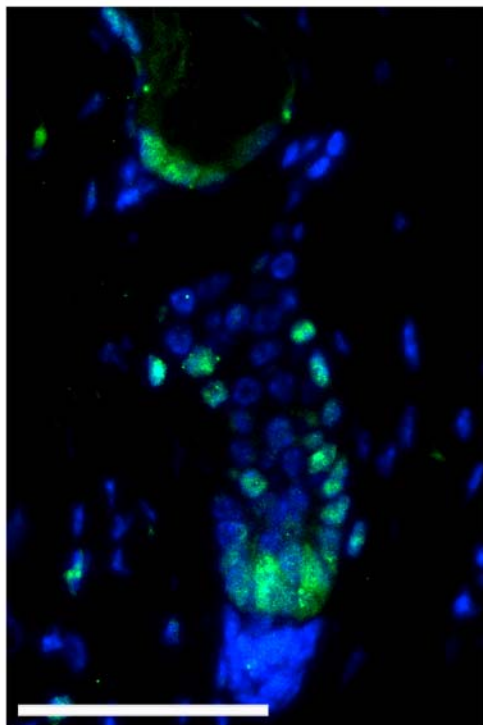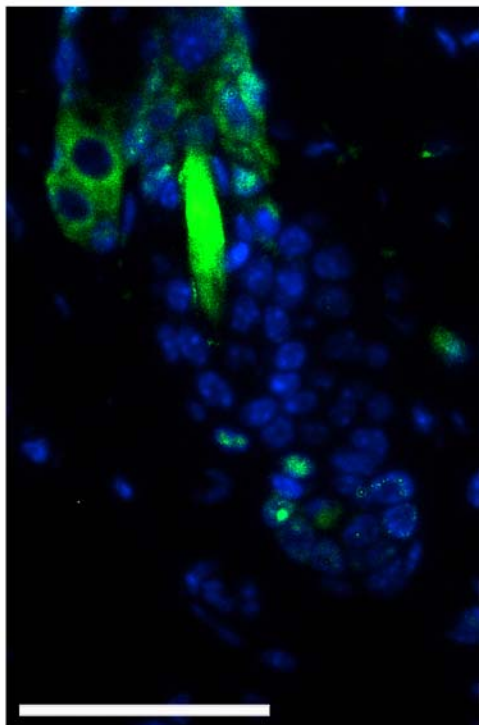

4d

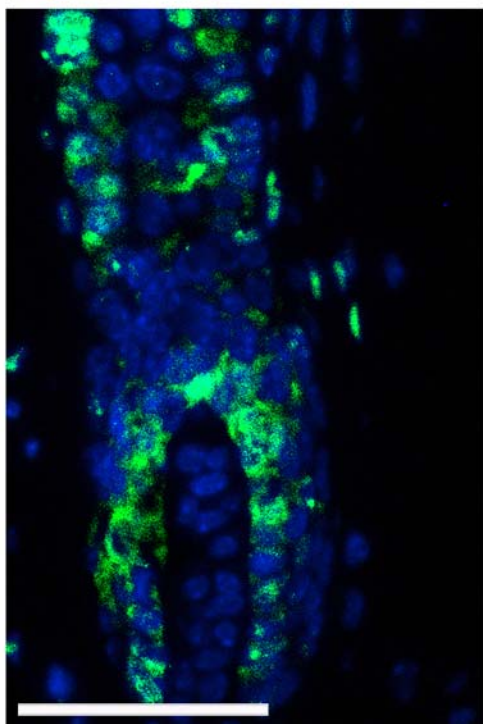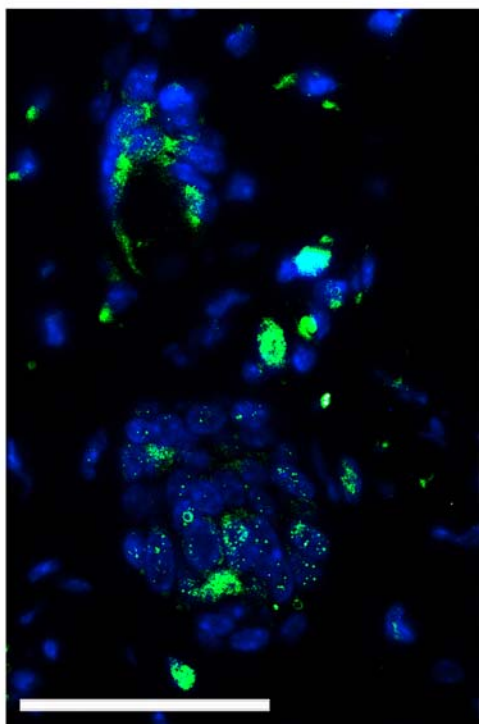

Supplement: Supplementary file 1 — In Supplementary Figure 1 and 2, the expression pattern of sFRP4 in mouse dorsal skin was detected by immunofluorescence. In Supplementary Figure 3, the effect of sFRP4 on the pigmentation of hair follicle was detected. The result showed that the skin eventually turned black when sFRP4 administration was ceased. In Supplementary Figure 4, the effect of sFRP4 on the differentiation of hair follicle cells was examined. The result showed that the differentiated cells were reduced after sFRP4 treatment. In Supplementary Figure 5, the effect of sFRP4 on the proliferation of hair follicle cells was examined. The result showed that the proliferated cells were reduced after sFRP4 treatment. [file 2857478.f1.zip › mat.2857478.v3 (1).pdf]
